# Dermatologic toxicities in epidermal growth factor receptor: a comprehensive pharmacovigilance study from 2013 to 2023

**DOI:** 10.3389/fmed.2023.1283807

**Published:** 2024-01-24

**Authors:** Hanyu Dan, Qiang Jiang, Xiangnan Jia, Guanpeng Qi, Dongsheng Zong, Zuojing Li

**Affiliations:** ^1^Medical Information Analysis Laboratory, College of Medical Devices, Shenyang Pharmaceutical University, Shenyang, China; ^2^School of Artificial Intelligence and Computer Science, Jiangnan University, Wuxi, China

**Keywords:** epidermal growth factor receptor inhibitors, cutaneous toxicity, pharmacovigilance, data mining, FAERS

## Abstract

Epidermal growth factor receptor inhibitors (EGFRIs) induced cutaneous toxicity is a common adverse event (AE), although it is not as severe as major cancers, we still need to pay enough attention to them. Therefore, it is necessary to evaluate the diversity of EGFRI class drugs. The objective of this study was to conduct a scientific and systematic investigation into the correlation between EGFRI and cutaneous toxicities. The data accessed from the FDA adverse event reporting system database (FAERS) encompass a time frame spanning from January 2013 to March 2023. By utilizing reporting odds ratios (RORs), information components (ICs), proportional reporting ratios (PRRs), and chi-squared (χ^2^), the relationship between drugs and adverse reactions was evaluated through disproportionality analysis. Within the FAERS database, a total of 29,559 skin adverse events were recorded. A robust indication of the correlation between EGFRI and elderly patients (≥65 years) was identified. Among EGFRIs, erlotinib accounted for the largest proportion of skin adverse events (39.72%). Rash, dry skin, and pruritus ranked top among all preferred terms, and signals such as rash, skin lesions, and acneiform dermatitis were detected in every single drug. Clinicians should guide patients customize the treatment plan for each patient.

## 1 Introduction

Epidermal growth factor receptor (EGFR) belongs to the receptor tyrosine kinase family (ErbB) that regulates tumor cell differentiation, survival, and proliferation. The EGFR family is also known as the EGFR tyrosine kinase family, which includes four receptor tyrosine kinases, such as EGFR/HER1, ErbB2/HER2, ErbB3/HER3, and ErbB4/HER4. Among them, EGFR is the first cell surface receptor found to be directly related to tumorigenesis. EGFR is also known as HER1, ErbB1, mutation, or overexpression generally cause tumors. Human epidermal growth factor receptor 2 (HER-2), a receptor that exists on the surface of breast cancer cells and is closely related to the occurrence and development of breast cancer (1–4). Epidermal growth factor receptor inhibitors (EGFRIs) are now well established as effective agents for the treatment of various cancers (5), which include monoclonal antibodies(mAbs): cetuximab, necitumumab, and panitumumab; tyrosine kinase inhibitors (TKIs): afatinib, dacomitinib, erlotinib, gefitinib, and Osimertinib; and Tyrosine multikinase inhibitors: lapatinib and vandetanib (6–8).

EGFRI has shown significant curative effect, changing the prospects for non-small cell lung cancer (NSCLC) (9), metastatic colorectal cancer (10), breast cancer (11), thyroid cancer (12), and rectal cancer (rectal cancer) (13). In the IPASS study, first-line treatment with gefitinib significantly prolonged progression-free survival in patients with lung adenocarcinoma compared with paclitaxel plus carboplatin (14). The National Comprehensive Cancer Network (NCCN) has recommended mAbs for the first-line treatment of patients with wild-type RAS metastatic colorectal cancer (mCRC) (15). EGFRI can also be combined with other drugs to treat cancer, such as the combination of EGFRI and immune checkpoint inhibitors (ICI) in the treatment of non-small cell lung cancer (NSCLC) (16).

However, a first-line cohort study of icotinib in 152 patients with EGFR-mutated advanced NSCLC reported a major safety profile, with rash and paronychia occurring in 43.4 and 5.9% of patients (17), respectively. The indications of EGFRIs continue to expand to different cancers and different stages of the disease. Skin diseases can be divided into early and late stages, with the former being better known and usually easier to prevent and treat. Later in treatment, other skin toxicities such as xerosis and eczema, with accompanying pruritus, may occur (18). And due to its specific mechanism of action, cutaneous toxicity has become the most common adverse drug reaction (ADR) of EGFRIs, which usually leads to a decrease in the quality of life of patients. Gefitinib-induced cutaneous toxicity led to dose discontinuation in 6.9% of patients (19). In one survey, 10% of patients admitted to skipping doses due to side effects (20). More attention and awareness of adverse event-induced EGFR inhibitor-related cutaneous adverse events is needed for prevention and treatment.

FAERS is a free and public voluntary database for collecting post-marketing drug adverse event information and is often used to carry out signal mining research on drug adverse events. The FAERS database can receive more than one adverse event information report about drugs and medical equipment every year. The adverse drug event information is spontaneously reported by drug manufacturers, hospital medical staff, pharmacists, and other professionals, as well as patients. Therefore, the information in the FAERS database is from the real world. Real-world safety data on EGFRI-related skin AEs are currently lacking, and there are inherent differences in activity and dermal toxicity among EGFRIs. This study outlines the safety profile of EGFRI through pharmacovigilance analysis and provides guidance for clinicians and patients so as to be familiar with how to prevent or improve skin AEs.

## 2 Methods

### 2.1 Data extraction

This study retrospectively mined and analyzed the skin toxicity AEs of EGFRI in FAERS from 1 January 2013 to 31 March 2023 through data mining, and a total of 41 quarterly report documents were screened, although a large proportion of these data comes from the United States, Europe, and the Asia-Pacific region, which also account for a large proportion. The following seven types of files make up the FAERS database: patient demographic and administrative information (DEMO), drug/biological information (DRUG), adverse events (REAC), patient outcomes (OUTC), reporting sources (RPSR), reporting drug therapy start and end dates (THER), and indications (INDI). These tables can be considered distinct domains. For instance, the tables within DEMO belonging to different years and quarters can be categorized as a single domain. Once the structure is standardized, tables from the same domain can be merged together. By integrating the data of specific years and quarters in each domain to facilitate subsequent management and analysis. In addition, there is a special file category named DELETED, which include the information about the CASEID of expurgated reports. These files exist in several certain quarters. All types of documents can be accessed from the FDA website.^1^

**Figure 1:**
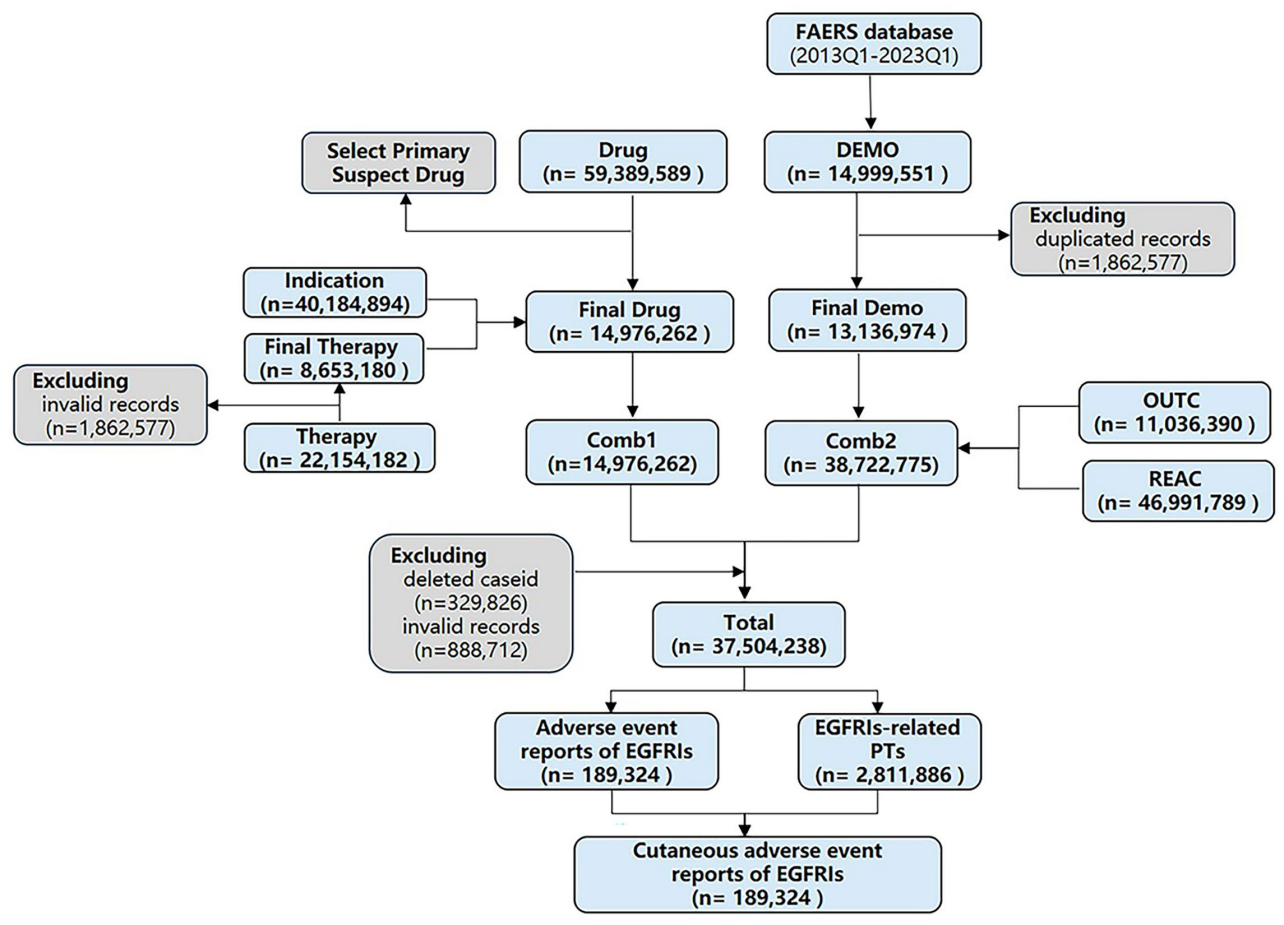
The flow diagram of selecting EGFRI-associated AEs from FAERS database.

According to the recommendation of FDA, we removed duplicate records by two steps: (1) selecting the greater PRIMARYID when the CASEID and EVENT_DT were the same and (2) selecting the latest EVENT_DT when the CASEID were the same. Then selected the drugs whose “ROLE_COD” fields are “PS” (Primary Suspect). Upon completing the screening process, we proceed with selecting six major file categories: DEMO, DRUG, REAC, OUTC, THER, and INDI. These categories serve as individual tables that can be linked through the common primary keys “PRIMARYID” and “CASEID” to consolidate patient and medication information. The DEMO, REAC, and OUTC tables are merged using the aforementioned primary keys. Additionally, the DRUG, INDI, and THER tables possess primary keys such as “DRUG_SEQ,” “INDI_DRUG_SEQ,” and “DSG_DRUG_SEQ,” respectively. Although these primary keys serve the same purpose, they have different variable names across the tables. By unifying the primary keys in these three tables, they can be merged together so that, the two large tables are merged into a single consolidated table for comprehensive analysis. This merging process is based on the two primary keys, “primaryid” and “caseid.” Finally, removed the wrongly uploaded report according to the CASEID in the DELETED folder (see Figure 1). SAS 9.4 (SAS Institute Inc., Cary, NC, United States) was used to integrate and process the raw data, and RStudio software was used to calculate the signal value for each group of clinical characteristics and visualize.

### 2.2 Target drugs and AEs

Our search in the FAERS database was conducted specifically for FDA-approved EGFRIs available on the market, encompassing mAbs (cetuximab, necitumumab, and panitumumab), TKIs (gefitinib, erlotinib, afatinib, dacomitinib, and osimertinib), VEGF/VEGFR inhibitors (vandetanib), and EGFR inhibitors/HER2 inhibitors (lapatinib) (Supplementary Table S1) by using trade and generic names listed in the National Center for Biotechnology Information (NCBI).

Adverse events with EGFRI-induced cutaneous toxicity in the FAERS database were defined as cases where the treatment regimen included drugs in the EGFRIs class and a skin-related adverse reaction in the SOC classification occurred. The SOC consists of six High Level Group Terms (HLGT), which includes epidermal and dermal conditions (MedDRA code 10014982), pigmentation disorders (MedDRA code 10035023), skin and subcutaneous tissue disorders NEC (MedDRA code 10040790), skin and subcutaneous tissue infections and infections (MedDRA code 10040792), skin appendage conditions (MedDRA code 10014982), and skin neoplasms malignant and unspecified (MedDRA code 10040785). All PTs were selected under the six HLGTs. Furthermore, the FAERS database recorded a single adverse event report for EGFRI in relation to cutaneous toxicity as one instance of data, despite the possibility of multiple adverse event reports being filed for the same patient, owing to the database’s structural and variable characteristics.

### 2.3 Time to onset

We analyzed the occurrence time of EGFRI-induced cutaneous toxicity. The occurrence time is the interval between START_DT (the time start therapy) and EVENT_DT (adverse event occurrence date). Incorrectly entered reports were excluded according to two exclusion criteria: (1) The value of START_DT or EVENT_DT is miss, (2) EVENT_DT is incorrect (START_DT later than EVENT_DT). The Kaplan–Meier curve can present the first quantile time, the median time, and the third quantile time and can be used to describe the changes in the incidence of AE. Discriminate the statistical difference in AE occurrence time between different EGFRI drug treatment regimens, mainly using the Kruskal–Wallis test and Wilcoxon rank-sum test to calculate.

### 2.4 Statistical analysis

#### 2.4.1 Disproportionality analysis

Disproportionality analysis is one of the most basic data mining methods for adverse drug reaction monitoring, which can also be called case-non-case analysis. Disproportionality analysis compared selected ADR proportions for a single drug or drug class with the same ADR proportions reported for other drug groups (21). There are two primary types of proportional imbalance methods that exist nowadays. One method is based on frequency, called the frequency method, and the other is based on Bayes’ theorem, also known as the Bayesian method. The former method, comprising the reporting odds ratio method and the proportional reporting ratio method, offers the advantages of low computational complexity, low time consumption, and independence from the need for *a priori* information in the model. Additionally, it exhibits resilience to non-selective underreporting of drugs or adverse drug reactions, thus not impacting the calculated ROR values in comparison with the patient population experiencing ADRs. However, it cannot be calculated when the denominator is zero, and it is easily affected by individual values. When the frequency is small, the statistics fluctuate greatly. The advantages and disadvantages of the PRR method are similar to those of the ROR method, but there is a fundamental difference. The ROR method calculates the statistic as the odds ratio (OR), while the PRR method calculates the relative hazard ratio (RR). The chi-squared test can be used in conjunction with the PRR method. The BCPNN method not only considers the information of probability asymmetry but also considers the information of the overall sample, which is more flexible and stable than the frequency method. In addition, since the model variable is not settled, the prior distribution corresponding to the variable will use a different distribution according to the change of the data, so there is no restriction on conditions for usage. Given the absence of a universally accepted “gold standard,” it is important to recognize that each method possesses its own unique set of advantages and disadvantages concerning its applicability and feasibility in varying scenarios. Therefore, in practical applications, the four methods should be combined to comprehensively evaluate the results of pharmacovigilance signals.

The detailed information for calculating the AE reports of the target drug and other drugs can be found in 2 × 2 Matrix Table (Table 1). In order to investigate potential correlations between EGFRI and cutaneous toxicities, four data mining methods were employed: the reporting odds ratio (ROR) method (22), proportional reporting rate (PRR) method, chi-squared (χ^2^) method (23), and the Bayesian confidence propagation neural network’s (BCPNN) information component (IC) method (24).

**Table 1:** 2*2 matrix table for EGFRI-induced dermatologic toxicities.

	Cutaneous toxicity-related reports	Non-cutaneous toxicity-related reports
Target drug-related reports	a	b
Not target drug	c	d

##### 2.4.1.1 Reporting odds ratio method

The ROR can be calculated using the following formula


$$\;ROR=\;\;\frac{a/c}{b/d}=\frac{ad}{bc}$$

Calculating the standard error of ln (ROR) and 95% confidence interval for the ROR involves the following steps


$$SE\left(lnROR\right)=\sqrt{\left(\frac{1}{a}+\frac{1}{b}+\frac{1}{c}+\frac{1}{d}\right)}$$

$$95\%CI=e^{\ln\left(ROR\right)\;\pm \;1\mathrm{.96}\sqrt{\frac{1}{a}+\frac{1}{b}+\frac{1}{c}+\frac{1}{d}}}$$

##### 2.4.1.2 Proportional reporting rate (PRR) method

The PRR measure can be expressed as


$$PRR=\frac{\left(a\right)/\left(a+b\right)}{\left(c\right)/\left(c+d\right)}$$

##### 2.4.1.3 Chi-squared (χ^2^) method

The χ^2^ can be expressed as


$$\chi^{2}=\sum \left(O-E\right)2/E$$

where *O* represent the observed count ( O=a ), and E represent the expected count [ E=(a+b)(a+c)/(a+b+c+d) ].

##### 2.4.1.4 BCPNN method

The calculation of its variance can be derived from Bayes’ theorem as


$$E\left(ICij\right)=\log_{2}\frac{\left(c_{ij+}\gamma_{ij}\right)\left(C+\alpha \right)\left(C+\beta \right)}{\left(C+\gamma \right)\left(c_{i}+\alpha_{i}\right)\left(C_{j}+\beta_{j}\right)}$$

$$V\left(ICij\right)=\frac{\begin{array}{l} \frac{C-c_{ij}+\gamma -\gamma_{ij}}{\left(C_{ij}+\gamma_{ij}\right)\left(1+C+\gamma \right)}+\frac{C-c_{i}+\alpha -\alpha_{i}}{\left(c_{i}+\alpha_{i}\right)\left(1+C+\alpha \right)}\\ +\frac{C-c_{j}+\beta -\beta_{i}\;}{\left(c_{i}+\beta_{j}\right)\left(1+C+\beta \right)} \end{array}}{{\left(\log2\right)}^{2}}$$

where


$$\gamma =\gamma_{ij}\frac{\left(C+\alpha \right)}{\left(C_{i}+\alpha_{i}\right)}\cdot \frac{\left(C+\beta \right)}{\left(C_{j}+\beta_{j}\right)}$$

and $\gamma_{ij}=1$ ; $\alpha_{i}=1$ , α=2 , $\beta_{j}=1$ , β=2 , *C* is the total number of reports in the database, *C_ij_* the number of combinations between an EGFRI drug (*i*) and the dermatologic toxicities drug reaction (*j*), *C_i_* the total number of reports on EGFRI drugs (*i*) in the database and *C_j_* the total number of reports on the dermatologic toxicities ADR (*j*) in the database.

The calculation of IC can be obtained as.


$$\;IC=\log_{2}\left[\frac{P\left(Drug|ADR\right)}{P\left(ADR\right)}\right]=\log_{2}\left[\frac{P\left(Drug,ADR\right)}{P\left(Drug\right)P\left(ADR\right).}\right]\;$$

There are diverse criteria for signal detection; ROR025 and IC025 represent the lower limit of the 95% confidence interval of ROR and IC. The signal was defined as positive if ROR025 was greater than 1 or IC025 was greater than 0. Besides, the signal was defined PRR is not less than 2 and chi-squared is not less than 4. Three or more cases should be met for all the criteria.

## 3 Results

### 3.1 Descriptive analysis

After processing, the FAERS database recorded a total of 37,504,238 data between 2013 and 2023. Among these records, a total of 189,324 adverse events attributed to EGFRIs were reported, with 29,559 reports specifically associated with cutaneous toxicity. Demographic information about the patients is summarized in Table 2.

**Table 2:** Demographic information of EGFRI-induced adverse events.

		Cutaneous toxicities AEs	Cutaneous toxicities AEs			
		With EGFRIs	With all other drug	IC025	ROR025	PRR (χ^2^)
		(29559)	(2978563)			
Sex	Male	10,805(36.55%)	775,936(27.89%)	**0.35**	**1.45**	1.31**(776.80)**
	Female	17,047(57.67%)	1,780,249(63.98%)	−0.18	0.75	0.90**(180.45)**
	TS	0(0.00%)	13(0.00%)			
	Unisex	0(0.00%)	20(0.00%)			
	Unknown	5(0.02%)	752(0.03%)			
	Miss*	1,589(5.33%)	216,711(7.68%)			
Age	Elderly	9,954(33.67%)	494,970(18.63%)	**0.81**	**2.16**	1.81**(3480.90)**
	Adult	8,846(29.92%)	1,230,517(43.96%)	−0.58	0.53	0.68**(1302.52)**
	Adolescent	14(0.05%)	111,167(3.97%)			
	Child	17(0.06%)	49,713(1.78%)			
	Infant	2(0.06%)	3,779(0.14%)			
	Neonate	0(0.00%)	2004(0.07%)			
	Miss*	10,726(36.29%)	844,142(31.44%)			
Outcome	Other Serious	11,501(38.91%)	865,428(31.10%)	**0.33**	**1.44**	1.25**(565.19)**
	Hospitalization	4,216(14.27%)	322,919(11.61%)	**0.24**	**1.22**	1.23**(175.60)**
	Life-threatening	414(1.40%)	29,162(1.04%)	**0.30**	**1.24**	1.34**(34.18)**
	Disability	340(1.15%)	46,099(1.66%)	−0.63	0.71	0.69**(44.97)**
	Death	1,518(5.18%)	32,041(1.15%)	**2.09**	**4.63**	**4.48(3846.70)**
	RI	32(0.11%)	4,893(0.18%)	−1.21	0.43	0.62**(7.55)**
	Congenital anomaly	20(0.07%)	1992(0.06%)	−1.77	0.29	0.95(0.06)
Country	US	17,042(57.32%)	1,872,444(67.30%)	−0.27	0.61	0.85**(466.01)**
	JP	3,399(11.20%)	49,843(1.79%)	**2.53**	**6.79**	**6.34(14164.07)**
	CN	856(2.90%)	19,696(0.71%)	**1.86**	**3.86**	**4.04(1857.719)**
	FR	637(2.16%)	76,531(2.75%)	−0.48	0.71	0.77**(41.24)**
	DE	569(1.93%)	53,631(1.93%)	−0.14	0.91	0.99(0.10)
	BR	566(1.91%)	30,742(1.10%)	**0.64**	**1.59**	1.71**(162.85)**
	CO	510(1.72%)	11,095(0.40%)	**1.91**	**3.96**	**4.27(1210.078)**
	IT	462(1.56%)	33,427(1.24%)	**0.18**	**1.14**	1.25**(22.21)**

IC, information component; IC025, the lower end of the 95% confidence interval of IC; ROR, reporting odds ratio; ROR025, the lower end of the 95% confidence interval of ROR; PRR, proportional reporting ratio; χ^2^, chi-squared. Bold values: index that meet the criteria for signal detection. *Unreported or lost reports.

In all reported cases of cutaneous toxicity induced by EGFRI, females accounted for a higher percentage than males (57.67% versus 36.65%). But by further analysis, signal was only detected in males (ROR025 = 1.45, IC025 = 0.35, PRR = 1.31, χ^2^ = 776.80). Significant differences were found among various subgroups in the study, indicating a greater proportion of elderly (> 65 years old, 33.67%) compared with non-elderly (29.92% for adults, 0.05% for adolescents, and 0.06% for children), and the difference is statistically significant (ROR025 = 2.16, IC025 = 0.81, PRR = 1.81, χ^2^ = 3480.90), which probably be attributed to degenerative changes in the elderly. Other serious medical events, hospitalization, and life-threatening emerged as the most commonly reported outcome events. Other serious medical events (ROR025 = 1.44, IC025 = 0.33, PRR = 1.25, χ^2^ = 569.19), hospitalization (ROR025 = 1.22, IC025 = 0.24, PRR = 1.23, χ^2^ = 175.60), life-threatening (ROR025 = 1.24, IC025 = 0.30, PRR = 1.34, χ^2^ = 34.18), and death (ROR025 = 2.09, IC025 = 4.63, PRR = 4.48, χ^2^ = 3846.70) indicate the life-threatening nature of potential EGFRI-related cutaneous toxicity. The United States (57.32%) represented the most frequently mentioned regions or countries, with Japan (11.20%), China (2.90%), France (2.16%), and Germany (1.93%) behind.

For all reports concerning EGFRI-related cutaneous toxicity, age and sex were analyzed separately for each class of EGFRI to investigate their relationship (Table 3). Signals were detected for all single agents in the elderly population, with a greater proportion of signals detected in males compared with females among all single drugs.

**Table 3:** The signals of cutaneous adverse events in EGFRI-related age and sex groups.

			Afatinib	Cetuximab	Dacomitinib	Erlotinib	Gefitinib	Lapatinib	Necitumumab	Osimertinib	Panitumumab	Vandetanib
Age group	Elderly	Count	1,501	1,388	71	3,548	475	773	47	935	1,590	120
		IC025	**1.26**	**0.81**	**0.12**	**0.58**	**1.30**	**0.15**	**0.69**	**1.11**	**0.90**	**0.20**
		ROR025	**3.69**	**2.19**	**1.48**	**1.71**	**4.08**	**1.21**	**2.37**	**3.09**	**2.41**	**1.28**
		PRR (χ^2^)	**2.53(1388.05)**	1.86**(550.06)**	1.93**(31.67)**	1.54**(669.17)**	**2.74(525.18)**	1.20**(26.17)**	**2.36(36.79)**	**2.32(699.79)**	1.97**(759.30)**	1.41**(14.26)**
	Adults	Count	1,098	1,648	119	2,137	275	991	37	499	1776	266
		IC025	−0.44	−0.17	**0.54**	−1.40	−0.76	**0.03**	−0.89	−1.06	−0.17	**0.19**
		ROR025	0.63	0.85	**1.85**	0.26	0.47	**1.10**	0.46	0.35	0.85	**1.50**
		PRR (χ^2^)	0.79**(58.91)**	0.95**(5.02)**	1.38**(12.76)**	0.40**(1951.25)**	0.68**(41.22)**	1.10**(10.23)**	0.80(1.92)	0.53**(207.52)**	0.94**(5.94)**	1.34**(22.94)**
Sex group	Male	Count	947	2,468	88	3,954	261	33	78	465	2,203	233
		IC025	**0.02**	**1.10**	**0.31**	**0.18**	−0.15	−4.56	**0.94**	−0.49	**0.83**	**0.70**
		ROR025	**1.05**	**4.05**	**1.60**	**1.21**	0.91	0.03	**4.73**	0.66	**2.64**	**2.47**
		PRR (χ^2^)	1.08**(5.21)**	**2.22(1647.55)**	1.60**(19.78)**	1.16**(83.13)**	1.02(0.15)	0.06**(507.43)**	**2.62(78.25)**	0.78**(30.07)**	1.84**(862.90)**	1.92**(107.40)**
	Female	Count	2,106	1,260	105	7,788	625	1879	19	1,533	1,466	160
		IC025	−0.02	−1.11	−0.61	−0.05	−0.05	**0.44**	−2.53	**0.06**	−0.98	−1.07
		ROR025	1.05	0.24	0.48	0.94	1.06	**6.13**	0.07	**1.30**	0.27	0.26
		PRR (χ^2^)	1.05**(4.98)**	0.50**(639.08)**	0.84(3.24)	1.00(0.01)	1.07(3.18)	1.45**(269.14)**	0.28**(35.02)**	1.13**(22.37)**	0.54**(578.82)**	0.56**(55.98)**

This table shows the signals of patients’ sex and age with dermatologic toxicities using EGFRIs. Count, number of records. ROR, reporting odds ratio; IC, information component; IC025, the lower limit of the 95% confidence interval of IC; ROR025, the lower end of the 95% confidence interval of ROR; PRR, proportional reporting ratio; χ^2^, chi-squared. Bold values: indexes that meet the criteria for signal detection.

Across all EGFRI regimens, discernible differences were identified in specific adverse events associated with cutaneous toxicity. The top reported reports were rash (8,040 cases, 27.20%), dry skin (1934 cases, 6.54%), itching (1722 cases, 5.83%), acne like dermatitis (1,274 cases, 4.31%), acne (1,198 cases, 4.05%), alopecia (1,102, 3.73%), paronychia (1,004, 3.40%), erythema (809, 2.74%), skin lesions (651, 2.20%), skin exfoliation (611, 2.07%), and skin toxicity (591, 2.00%) (Table 4). Collectively, these comprised 64.07% of the total.

**Table 4:** Distribution of dermatologic toxicities related to EGFRIs drugs.

Dermatologic toxicities	*N*	Percentage
Rash	8,040	27.20
Dry skin	1934	6.54
Pruritus	1722	5.83
Dermatitis acneiform	1,274	4.31
Acne	1,198	4.09
Alopecia	1,102	3.73
Paronychia	1,004	3.40
Erythema	809	2.74
Skin disorder	651	2.22
Skin exfoliation	611	2.07
Skin toxicity	591	2.00
Others	10,390	35.48

### 3.2 Cutaneous toxicity AE profile in treatment protocols

Overall, while not all EGFRIs exhibited associations with cutaneous AEs, signals were identified when analyzing each drug individually in relation to cutaneous toxicity AEs. Among the analysis of all EGFRIs and individual EGFRIs, erlotinib and panitumumab similarly demonstrated the most robust statistical association with EGFRI-related cutaneous AEs according to the value of IC025, ROR025, and PRR (χ^2^). Panitumumab has higher IC025, ROR025, and PRR than erlotinib, but erlotinib has more counts and higher χ^2^ than panitumumab (Table 5).

**Table 5:** Signals for overall and each class of EGFRIs drugs with dermatologic toxicity AEs.

	(a)	(b)	(c)	(d)	ROR025	IC025	PRR(χ^2^)
Total	29,559	159,765	2,782,327	34,858,945	**2.29**	**1.05**	**2.11 (17043.8)**
Afatinib	3,157	19,586	2,810,304	34,997,549	**1.93**	**0.85**	1.87**(1269.95)**
Cetuximab	3,974	21,361	2,807,912	34,997,359	**2.24**	**1.03**	**2.11 (2321.60)**
Dacomitinib	196	1,274	2,811,806	35,017,320	**1.65**	**0.62**	1.79**(68.85)**
Erlotinib	12,267	57,154	2,807,667	34,953,508	**2.62**	**1.22**	**2.38 (9720.43)**
Gefitinib	922	9,345	2,811,142	35,009,187	**1.15**	**0.17**	1.21**(33.05)**
Lapatinib	2,043	13,185	2,810,833	35,004,535	**1.85**	**0.78**	1.80**(732.53)**
Necitumumab	106	512	2,811,849	35,018,129	**2.09**	**0.88**	**2.31 (78.54)**
Osimertinib	2,147	29,111	2,809,648	34,989,690	1.04	−0.01	1.01**(**1.07**)**
Panitumumab	4,294	19,167	2,810,531	34,996,604	**2.70**	**1.25**	**2.46 (3720.20)**
Vandetanib	453	3,130	2,811,669	35,015,344	**1.63**	**0.62**	1.70**(130.82)**

(a) The number of skin AEs reported for EGFRIs. (b) The number of any other AEs reported for EGFRIs. (c) The number of any skin AEs for other drugs. (d) The number of other AEs reported for other drugs. ROR, reporting odds ratio; IC, information component; IC025, the lower limit of the 95% confidence interval of IC; ROR025, the lower end of the 95% confidence interval of ROR; PRR, proportional reporting ratio; χ^2^, chi-squared. Bold values: indexes that meet the criteria for signal detection.

**Figure 2:**
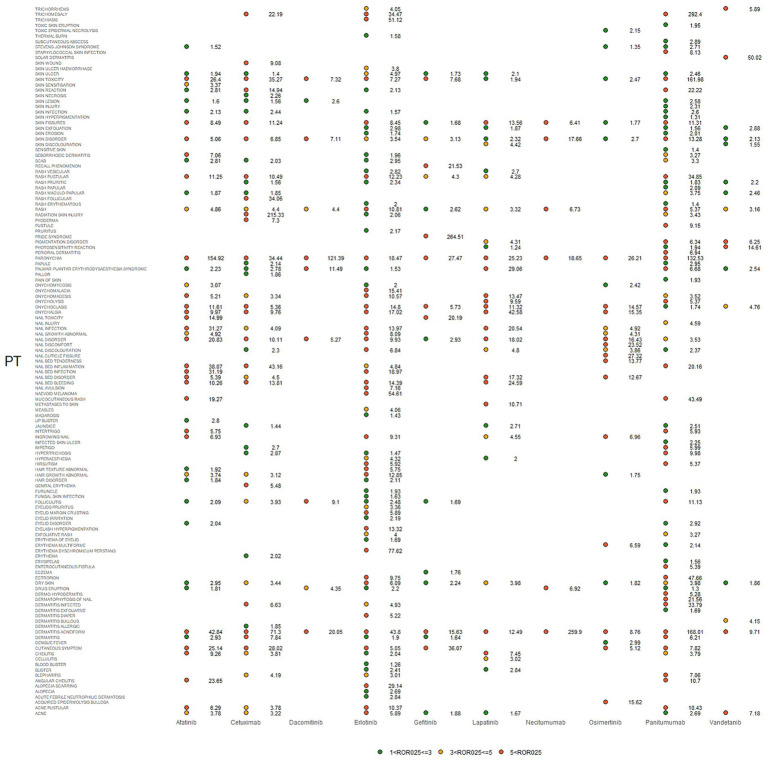
Adverse events (AEs) related to dermatologic toxicity associated with epidermal growth factor inhibitor (EGFRI) drugs exhibited signals indicated by reporting odds ratios (RORs). PT, preferred term. The lower end of the 95% confidence interval of ROR, denoted as ROR025, served as a threshold for identifying signals. ROR025 greater than 1 was deemed a signal.

**Figure 3:**
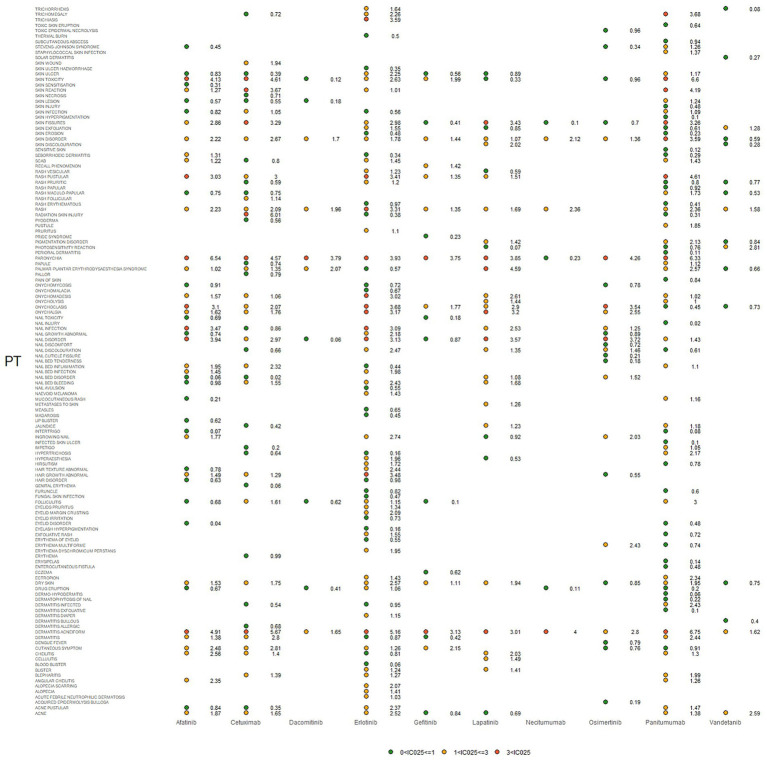
Signals were observed for epidermal growth factor inhibitor (EGFRI) drugs in relation to detailed adverse events (AEs) associated with dermatologic toxicity. PT, preferred term. The lower end of the 95% confidence interval of IC, denoted as IC025, served as the threshold for identifying signals. IC025 greater than 0 was deemed a signal.

**Figure 4:**
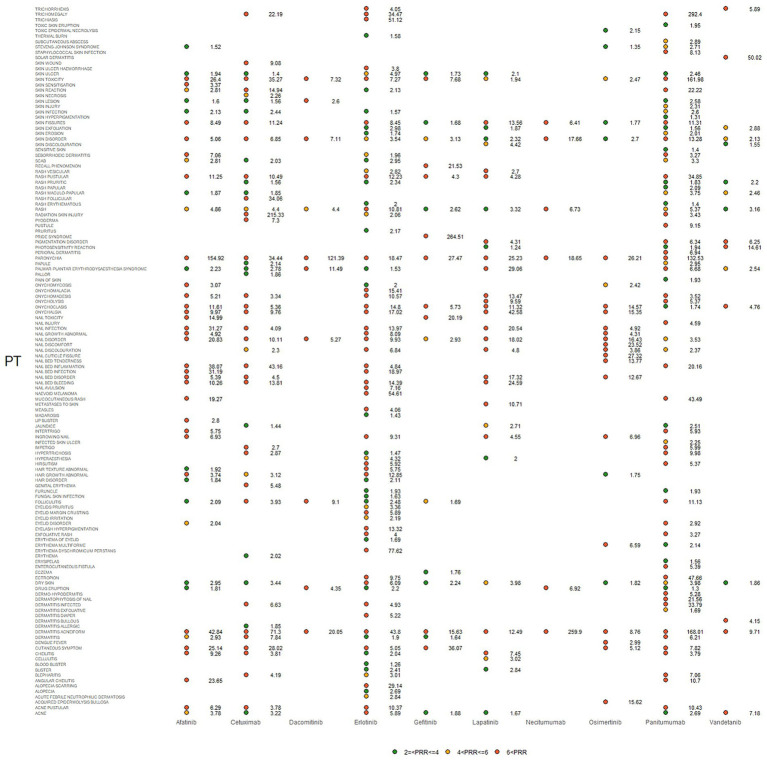
Signals were observed for epidermal growth factor inhibitor (EGFRI) drugs in relation to detailed adverse events (AEs) associated with dermal toxicity. PT, preferred term; PRR not less than 2 was deemed a signal.

IC025, PRR, and ROR025 between PTs for drugs and adverse events are depicted in Figures 2–4, respectively. Erlotinib had the broadest range of cutaneous AEs, with 74 PTs monitored for signals ranging from blood blister (IC025 = 0.06, ROR025 = 1.26, PRR = 2.53) to dermatitis acneiform (IC025 = 5.16, ROR025 = 43.80, PRR =48.64). A total of 51 PTs were found to be significantly associated with afatinib treatment, ranging from eyelid disorder (IC025 = 0.04, ROR025 = 20.3, PRR = 5.44) to paronychia (IC025 = 6.83, ROR025 = 221.43, PRR = 171.51). A total of 48 PTs were found to be significantly associated with cetuximab, ranging from nail bed disorder (IC025 = 0.02, R0R025 = 4.50, PRR = 14.03) to radiation skin injury (IC025 = 6.09, R0R025 = 264.61, PRR = 260.27). A total of 70 PTs were monitored as signals for panitumumab ranging from skin hyperpigmentation (IC025 = 0.10, R0R025 = 1.31, PRR = 2.63) to dermatitis acneiform (IC025 = 6.74, R0R025 = 168.01, PRR = 182.12). Lapatinib had 32 PTs monitored as signals ranging from photosensitivity reaction (IC025 = 0.07, ROR025 = 1.24, PRR = 2.38) to onychalgia (IC025 = 3.23, ROR025 = 47.70, PRR = 67.91). Osimertinib had 25 PTs monitored as signals ranging from Stevens–Johnson syndrome (IC025 = 0.34, ROR025 = 1.36, PRR = 2.10) to paronychia (IC025 = 4.59, ROR025 = 36.50, PRR = 32.12). And PTs detected in gefitinib, vandetanib, dacomitinib, and necitumumab were 21, 16, 11, and 6, respectively. Signs of rashes, skin lesions, and acneiform dermatitis were detected in all drugs.

### 3.3 Time to onset

**Figure 5:**
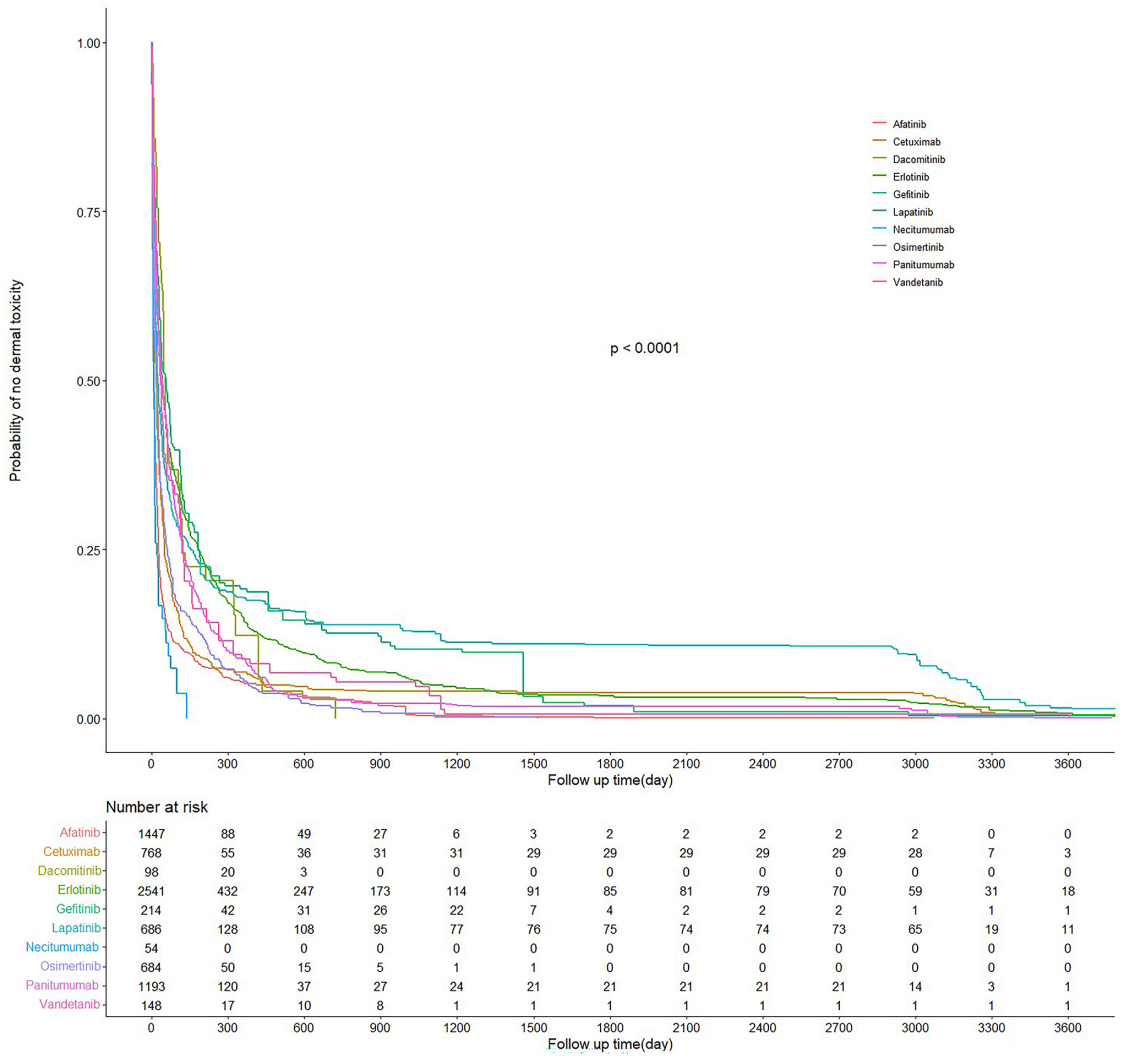
AE reports of EGFRI-induced dermatologic toxicities in Kaplan–Meier curves and risk tables.

In total, 7,933 EGFRI-related cutaneous toxicity AEs were reported. Displayed in Figure 5 are the Kaplan–Meier curves, illustrating the onset time of adverse events (AEs) for different EGFRIs. The corresponding risk table, situated at the bottom of Figure 5, presents the number of individuals followed at each time point. Significantly different AE onset times among the various EGFRIs were observed following the Kruskal–Wallis test, with a value of p of less than 0.0001.

The median time to onset was 25 days, accompanied by an interquartile range (IQR) spanning from 7 to 108 days. Detailed information regarding the time to onset of cutaneous toxicity AEs for each specific EGFRI can be found in Table 6. Necitumumab exhibited the shortest median time to AE onset, recorded at 9 days with IQR of 5–16 days. Conversely, gefitinib demonstrated the longest median time to AE onset, reported as 57 days, with an IQR of 14–182 days.

**Table 6:** The occurrence time of skin AEs for EGFRIs.

	Q1	Median	Q3	IQR
Afatinib	4	9	28	24
Cetuximab	7	19	50	43
Dacomitinib	27	54	123	96
Erlotinib	11	42	186	175
Gefitinib	14	57	182	168
Lapatinib	7	24	154	147
Necitumumab	5	9	16	11
Osimertinib	7	22	64	57
Panitumumab	13	42	125	112
Vandetanib	13	38	119	106

Q1 refer to the first quartile time, Q3 refer to the third quartile time, all values are in days.

**Figure 6:**
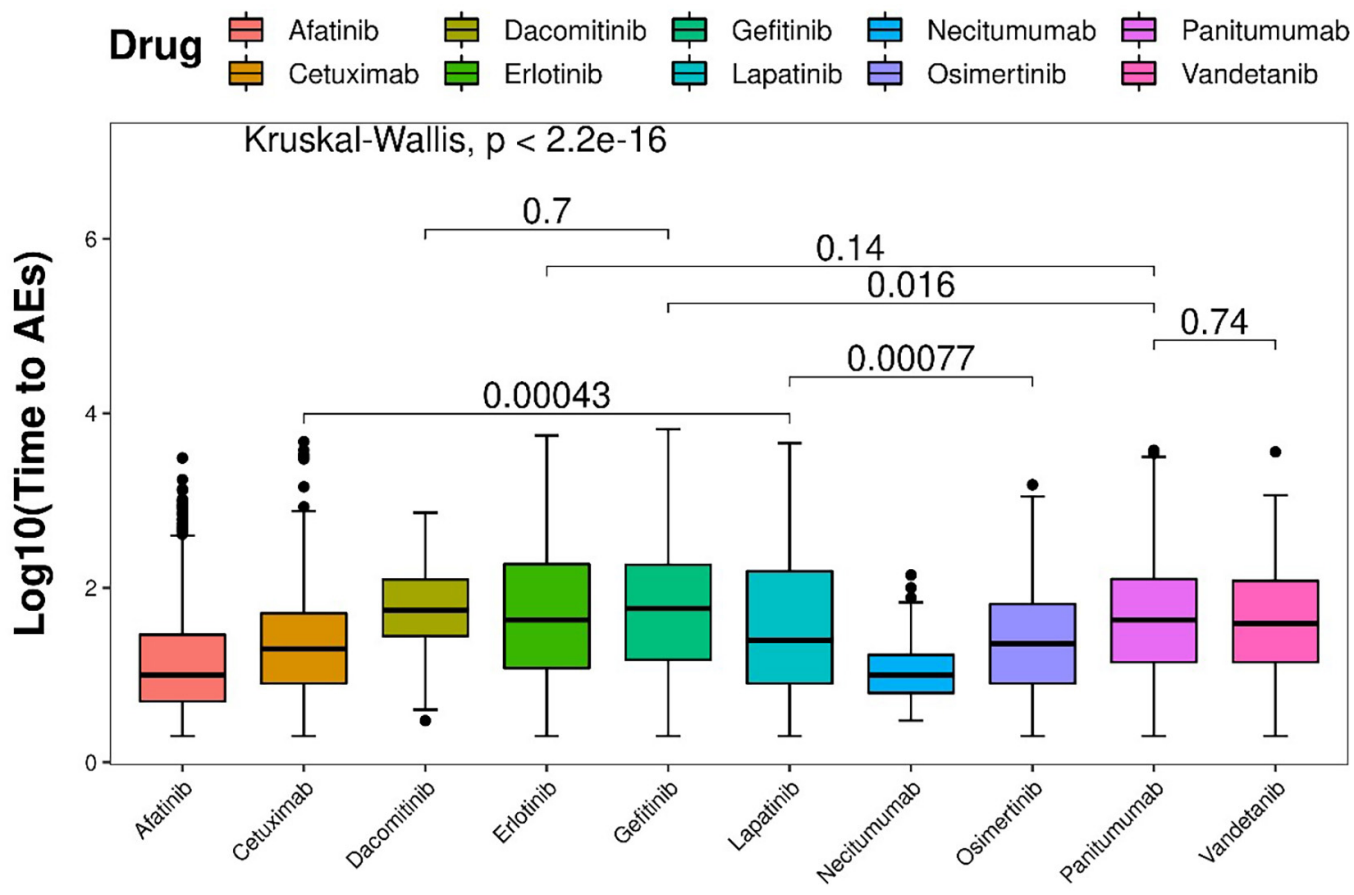
Time interval disparities analysis between durations of EGFRIs-related dermatologic AEs.

Based on the Wilcoxon rank-sum test, pairwise comparisons were conducted to assess difference among different drugs. In terms of the time to onset of AEs, panitumumab demonstrated a significantly shorter duration than gefitinib, but not significantly shorter than vandetanib and erlotinib. Conversely, lapatinib was significantly shorter than osimertinib and cetuximab. As for dacomitinib, it did not show a significant difference compared with erlotinib (Figure 6).

## 4 Discussion

We analyzed EGFRI-related adverse events in the FAERS database by ROR, PRR, chi-squared, and BCPNN methods and identified associations and specificities between EGFRI and related skin toxicity AEs to delineate the safety profile. Launch dates for EGFRIs vary, with gefitinib first being on the market in 2003. However, based on clinical use, second- and third-generation tyrosine kinase inhibitors are currently the most used and widely available, which are listed after 2012. Redundant data in the analysis amplify the likelihood of probability errors in the results. Encompassing both currently utilized and previously marketed EGFRIs, we conducted pharmacovigilance studies utilized over 30 million records to investigate EGFRI-related skin toxicity within a specific time period.

Most clinical trials of EGFRIs assessed clinical outcomes, although some assessed AEs but not in sufficient detail, and only the grading degree and brief descriptions of these AEs have been provided. The published phase III studies did not include detailed information on the prevention of cutaneous toxicity. On the other side, the impact on pathology-related QoL is so far unclear. Prior investigations have presented evidence indicating that EGFRI escalates the risk of toxicity at the organ system level, encompassing pulmonary toxicity (25)and cardiotoxicity (26).

The analysis of AE time intervals reveals that for most EGFRI-related skin toxicity AEs, they occur within the first few days to 2 months after administration. Subsequently, based on the Kaplan–Meier curves, the incidence rate gradually descends. Adverse events persist in patients throughout the subsequent treatment, extending for months or even years (27). The median time from post-administration to AE occurrence, representing the point at which the AE incidence reaches 50%, was similar for cetuximab, lapatinib, and osimertinib, occurring on day 7 after administration. However, considering the third quartile time, which indicates the time required for AE incidence to reach 75% after administration, cetuximab required 50 days, osimertinib took 64 days, and lapatinib necessitated the longest duration at 154 days. It signifies that the risk of skin-related AEs in long-term use of lapatinib is low, possibly owing to its metabolic mechanism. Lapatinib has a broad metabolic distribution, and although multiple metabolite forms are excreted, only one can inhibit EGFR (28). Among mAbs, panitumumab exhibited the longest duration, the median time to AE onset was 42 days for panitumumab and cetuximab had shorter median times of 9 and 19 days, respectively. This observation may be attributed to the fact that panitumumab belongs to the IgG2 isotype and primarily functions by blocking EGFR without participating in immune mediation. On the contrary, IgG type 1 antibodies like cetuximab mediate cytotoxicity mediated by cell that is antibody-dependent (29, 30). In addition, despite being the latest mAbs drug selected for this study, necitumumab exhibited the earliest onset time of AEs, with the first quartile time observed on day 5 after administration, and the third quartile time recorded on day 16. Remarkably, the elder had a significant signal associated with cutaneous toxicity AEs (IC 025 = 0.81, ROR 025 = 2.16, PRR = 1.81, χ^2^ = 3480.90). The studies indicate an increased susceptibility among the elder to the development of skin AEs after treatment with EGFRI. According to previous investigations, more frequent and severe cutaneous toxicity was observed in elderly patients (31), which may be due to differences in pharmacogenomics or pharmacodynamics in the elderly. EGFRI drugs are mainly metabolized by CYP3A4, CYP3A5, and CYP1A1 in the cytochrome P450 enzyme family (32, 33), age-related changes may impact these metabolic pathways, leading to decreased activity observed in some elderly (34, 35). Patients taking EGFRI drugs, which are predominantly prescribed for NSCLC, may exhibit gender differences that are influenced by their distinct indications, EGFR has a greater mutation probability in female with NSCLC (36, 37).

In addition, rash and acneiform dermatitis had the strongest adverse reaction signal and the most widespread distribution. EGFRI-induced rashes vary in severity. The frequency and severity of rashes increased with antibodies compared with low-molecular-weight TKIs, which can be attributed to the process of antibody-mediated receptor internalization. This is a pivotal mechanism to abolish activation of receptor and may lead to enhanced inhibition of the EGFR signaling pathway (38). Dermatitis acneiform, characterized as a major cutaneous toxicity event, exhibits a signal across 10 drugs, with the most pronounced signal observed for panitumumab (IC025 = 6.75, ROR025 = 168.01, PRR = 182.12, χ^2^ = 68808.86). Controversially, previous studies using several different EGFR drugs have demonstrated a link between rash and clinical efficacy. Skin rash appears to be a surrogate marker of clinical benefit (39). However, as described elsewhere, additional analyzes are required to describe the correlation between rash occurrence and overall treatment response (40). In addition to the commonly mentioned adverse reactions in the instructions of EGFRI drugs, some new signals have been identified. Afatinib and erlotinib have been associated with drug eruptions, while cetuximab, erlotinib, and panitumumab have been linked to skin radiation lesions. Furthermore, lip blistering has emerged as a new signal for afatinib and cheilitis for cetuximab. Gefitinib has been linked to PRIDE syndrome and recall phenomenon. Osimertinib has been associated with toxic epidermal necrolysis and epidermolysis bullosa acquired 3. Panitumumab has been associated with intestinal fistula and perioral dermatitis. However, the cases of these signals are rare and the influence of other drugs or alternative therapies should be considered.

The mechanism by which EGFRI induces skin irritation has been described differently. Low-molecular-weight TKIs block phosphorylation of the cytoplasmic domain, while mAbs competitively inhibit ligand binding to the extracellular domain, exerting their activity (41). Drug-mediated blockade of EGFR leads to growth arrest and apoptosis in EGFR-dependent cells for survival by inhibiting downstream pathways, including mitogen-activated protein kinase pathway, MAPK for Abbreviation, phosphatidylinositol 3-kinase-Protein Kinase B pathway, PI3K-Akt for Abbreviation. And there are two pathways involving the stress-activated protein kinase pathway, one is protein kinase C, and the other is Janus kinase-signal transducer and activator of transcription, Jak–STAT for Abbreviation (42). Some skin AEs are considered to be triggered by impact of EGFR on basal keratinocytes. Suppression of signaling pathways mediated by EGFR exerts multiple effects on keratinocytes. It results in growth arrest and apoptosis induction, decreasing cell migration, enhancing cell attachment and differentiation, and triggering inflammation (43). ERK 1 (extracellular signal-regulated kinase 1) and ERK 2 were found to mediate releasing cytokine of epithelial cells (41). This may coordinate the recruiting and activating of leukocyte, including neutrophils, lymphocytes, and monocytes, With the increased release of effector cytokines, chemokine production is amplified and leukocyte recruitment is enhanced, triggering the occurrence of papulopustular rash and paronychia as a consequence (44). Collectively, the aforementioned studies provide evidence supporting the pathogenesis of skin rash and acneiform dermatitis.

Analyzing spontaneous reporting systems is a useful way to identify potential signals, and the FAERS database is one of the largest sources of data. However, our study currently has the following limitations: First, the accurate assessment of an event can be influenced by the variability in information completeness across different reports. And FAERS post-marketing data are spontaneous reports and cannot fully reflect the incidence of adverse events. Second, filling in the information in the FAERS database is affected by the patient’s subjective wishes. For example, the current condition is relatively mild, and some patients may not choose to report. In addition, due to the lack of follow-up/censored data, only the correlation between EGFRI and cutaneous adverse events can be determined, and it is difficult to establish causality. Finally, only cutaneous toxicities have been explored, the link of EGRFI to other organ systems has not been explored furthermore. In the future, we will compare other system organ class AE signals of EGFRIs to strengthen the study.

## 5 Conclusion

The correlation between EGFRI and skin AEs was thoroughly evaluated using the FAERS database and data mining methods in this study. Overall, significant associations were detected between EGFRIs and cutaneous AEs, with relatively notable signals for several of the EGFRI drugs. Part of the results align with prior research. Rash and acneiform dermatitis exhibited an association with all drugs, and paronychia was associated with most drugs (except vandetanib). As a clinician, when using EGFRIs clinically, it is necessary to educate and empower patients, especially elderly patients, to prevent and report toxicity in a timely manner, pay attention to the development of cutaneous toxicity AE and intervene when necessary. In particular, rash, an adverse reaction that may be underrecognized and undertreated, should be discussed with the patient about how the rash affects quality of life and how to manage it appropriately. These more common adverse reactions require communication and evaluation with the patient. To determine whether it is necessary to stop the drug or take other measures. In addition to rash, other skin-related AEs should be paid more attention to. Although there are fewer newly discovered signal cases, they still need to be paid attention to. Customizing a specific treatment plan for each patient is essential due to the variations in the onset and duration of each EGFRI drug, as well as the differences in AEs observed across different various groups.

## Data availability statement

The raw data supporting the conclusions of this article will be made available by the authors, without undue reservation.

## Ethics statement

Ethical approval was not required for the study involving humans in accordance with the local legislation and institutional requirements. Written informed consent to participate in this study was not required from the participants or the participants’ legal guardians/next of kin in accordance with the national legislation and the institutional requirements.

## Author contributions

HD: Conceptualization, Data curation, Formal analysis, Funding acquisition, Investigation, Methodology, Project administration, Resources, Software, Supervision, Validation, Visualization, Writing – original draft. QJ: Writing – original draft, Data curation, Formal analysis. XJ: Writing – original draft, Data curation, Formal analysis. GQ: Software, Visualization, Writing – original draft. DZ: Writing – review & editing. ZL: Writing – review & editing, Conceptualization.

## Footnotes

^1^https://fis.fda.gov/extensions/FPD-QDE-FAERS/FPD-QDE-FAERS.html

## References

[1] Yarden Y Sliwkowski MX. Untangling the ErbB signalling network. Mol Cell Biol. (2001) 2:127–37. doi: 10.1038/35052073 11252954

[2] Cohen SJC. The epidermal growth factor (EGF). Cancer. (1983) 51:1787–91. doi: 10.1002/1097-0142(19830515)51:10<1787::AID-CNCR2820511004>3.0.CO;2-A, PMID: 6299497

[3] Thompson D Gill GN. The EGF receptor: structure, regulation and potential role in malignancy. Cancer Surv. (1985) 4:767–88. 2824044

[4] Olayioye MA Neve RM Lane HA Hynes NE. The ErbB signaling network: receptor heterodimerization in development and cancer. EMBO J. (2000) 19:3159–67. doi: 10.1093/emboj/19.13.3159, PMID: 10880430 PMC313958

[5] Peréz-Soler R Saltz L. Cutaneous adverse effects with HER1/EGFR-targeted agents: is there a silver lining? JCO. (2005) 23:5235–46. doi: 10.1200/JCO.2005.00.6916, PMID: 16051966

[6] Reichert JM Dhimolea E. The future of antibodies as cancer drugs. Drug Discov Today. (2012) 17:954–63. doi: 10.1016/j.drudis.2012.04.006 22561895

[7] Peng Y Li Q Zhang J Shen W Zhang X Sun C. Update review of skin adverse events during treatment of lung cancer and colorectal carcinoma with epidermal growth receptor factor inhibitors. BST. (2018) 12:537–52. doi: 10.5582/bst.2018.01246, PMID: 30555112

[8] Zhou Y Zhang Y Zou H Cai N Chen X Xu L. The multi-targeted tyrosine kinase inhibitor vandetanib plays a bifunctional role in non-small cell lung cancer cells. Sci Rep. (2015) 5:8629. doi: 10.1038/srep08629, PMID: 25720956 PMC4342569

[9] GJJOCO G. Epidermal growth factor receptor inhibitors in the treatment of non–small-cell lung cancer. J Clin Oncol. (2005) 23:3235–42. doi: 10.1200/JCO.2005.08.409 15886311

[10] Bokemeyer C Bondarenko I Hartmann J De Braud F Schuch G Zubel A. Efficacy according to biomarker status of cetuximab plus FOLFOX-4 as first-line treatment for metastatic colorectal cancer: the OPUS study. Ann Oncol. (2011) 22:1535–46. doi: 10.1093/annonc/mdq632, PMID: 21228335

[11] Awada A Saliba W Bozovic-Spasojevic I. Lapatinib ditosylate: expanding therapeutic options for receptor tyrosine-protein kinase erbB-2-positive breast cancer. Drugs Today (Barc). (2011) 47:335–45. doi: 10.1358/dot.2011.47.5.1584110, PMID: 22013564

[12] Liebner DA Shah MH. Thyroid cancer: pathogenesis and targeted therapy. Ther Adv Endocrinol Metab. (2011) 2:173–95. doi: 10.1177/2042018811419889, PMID: 23148184 PMC3474640

[13] Willett CG Duda DG Czito BG Bendell JC Clark JW Jain RKJO. Targeted therapy in rectal cancer. Oncology (Williston Park). (2007) 21:1055. 17910311 PMC2686129

[14] Mok TS Wu Y-L Thongprasert S Yang C-H Chu D-T Saijo N. Gefitinib or carboplatin–paclitaxel in pulmonary adenocarcinoma. N Engl J Med Overseas Ed. (2009) 361:947–57. doi: 10.1056/NEJMoa0810699 19692680

[15] National Comprehensive Cancer Network. NCCN clinical practice guidelines in oncology: head and neck cancers. (2018). 10.6004/jnccn.2020.0031 32634781

[16] Milanezi F Carvalho S Schmitt FC. EGFR/HER2 in breast cancer: a biological approach for molecular diagnosis and therapy. Expert Rev Mol Diagn. (2008) 8:417–34. doi: 10.1586/14737159.8.4.417, PMID: 18598224

[17] Huang A Shen Q Yu X Wang H Shi C Han B. Efficacy, safety and prognostic factors analysis of first-line icotinib treatment in advanced non-small cell lung cancer patients with mutated EGFR. TCR. (2018) 7:600–8. doi: 10.21037/tcr.2018.05.30

[18] Hofheinz R-D Segaert S Safont MJ Demonty G Prenen H. Management of adverse events during treatment of gastrointestinal cancers with epidermal growth factor inhibitors. Crit Rev Oncol Hematol. (2017) 114:102–13. doi: 10.1016/j.critrevonc.2017.03.032 28477738

[19] Patil VM Noronha V Joshi A Choughule AB Bhattacharjee A Kumar R. Phase III study of gefitinib or pemetrexed with carboplatin in EGFR-mutated advanced lung adenocarcinoma. ESMO Open. (2017) 2:e000168. doi: 10.1136/esmoopen-2017-000168, PMID: 28761735 PMC5519810

[20] Anderson KR Chambers CR Lam N Yau PS Cusano F Savoie ML. Medication adherence among adults prescribed imatinib, dasatinib, or nilotinib for the treatment of chronic myeloid leukemia. J Oncol Pharm Pract. (2015) 21:19–25. doi: 10.1177/1078155213520261, PMID: 24503243

[21] Hauben M Bate A. Decision support methods for the detection of adverse events in post-marketing data. Drug Discov Today. (2009) 14:343–57. doi: 10.1016/j.drudis.2008.12.012 19187799

[22] Norén GN Hopstadius J Bate A. Shrinkage observed-to-expected ratios for robust and transparent large-scale pattern discovery. Stat Methods Med Res. (2013) 22:57–69. doi: 10.1177/0962280211403604, PMID: 21705438 PMC6331976

[23] Evans SJ Waller PC Davis SJP. Use of proportional reporting ratios (PRRs) for signal generation from spontaneous adverse drug reaction reports. Pharmacoepidemiol Drug Saf. (2001) 10:483–6. doi: 10.1002/pds.677, PMID: 11828828

[24] Stricker BHC Tijssen JGP. Serum sickness-like reactions to cefaclor. J Clin Epidemiol. (1992) 45:1177–84. doi: 10.1016/0895-4356(92)90158-J 1474414

[25] Bate A Evans SJP. Quantitative signal detection using spontaneous ADR reporting. Pharmacoepidemiol Drug Saf. (2009) 18:427–36. doi: 10.1002/pds.1742 19358225

[26] Xie X Wang X Wu S Yang H Liu J Chen H. Fatal toxic effects related to EGFR tyrosine kinase inhibitors based on 53 cohorts with 9,569 participants. J Thorac Dis. (2020) 12:4057–69. doi: 10.21037/jtd-19-4000A, PMID: 32944317 PMC7475571

[27] Kunimasa K Kamada R Oka T Oboshi M Kimura M Inoue T. Cardiac adverse events in EGFR-mutated non-small cell lung cancer treated with Osimertinib. JACC CardioOncology. (2020) 2:1–10. doi: 10.1016/j.jaccao.2020.02.003, PMID: 34396203 PMC8352275

[28] Wacker B Nagrani T Weinberg J Witt K Clark G Cagnoni PJJCCR. Correlation between development of rash and efficacy in patients treated with the epidermal growth factor receptor tyrosine kinase inhibitor Erlotinib in two large phase III studies. Clin Cancer Res. (2007) 13:3913–21. doi: 10.1158/1078-0432.CCR-06-2610, PMID: 17606725

[29] Lacouture M Laabs S Koehler M Sweetman R Preston A di Leo A. Analysis of dermatologic events in patients with cancer treated with lapatinib. Breast Cancer Res Treat. (2009) 114:485–93. doi: 10.1007/s10549-008-0020-7, PMID: 18600445

[30] Yang X-D Jia X-C Corvalan JR Wang P. Development of ABX-EGF, a fully human anti-EGF receptor monoclonal antibody, for cancer therapy. Crit Rev Oncol Hematol. (2001) 38:17–23. doi: 10.1016/S1040-8428(00)00134-7 11255078

[31] Malik I Hecht JR Patnaik A Venook A Berlin J Croghan G. Safety and efficacy of panitumumab monotherapy in patients with metastatic colorectal cancer (mCRC). J Clin Oncol. (2005) 23:3520. doi: 10.1200/jco.2005.23.16_suppl.3520

[32] Wheatley-Price P Ding K Seymour L Clark GM Shepherd FA. Erlotinib for advanced non–small-cell lung cancer in the elderly: an analysis of the National Cancer Institute of Canada clinical trials group study BR.21. J Clin Oncol. (2008) 26:2350–7. doi: 10.1200/JCO.2007.15.2280 18467727

[33] Li J Zhao M He P Hidalgo M Baker SDJCCR. Differential metabolism of Gefitinib and Erlotinib by human cytochrome P450 enzymes. Clin Cancer Res. (2007) 13:3731–7. doi: 10.1158/1078-0432.CCR-07-0088, PMID: 17575239

[34] Ling J Johnson KA Miao Z Rakhit A Pantze MP Hamilton M. Metabolism and excretion of ERLOTINIB, A small molecule inhibitor of epidermal growth factor receptor tyrosine kinase, in healthy male volunteers. Drug Metab Dispos. (2006) 34:420–6. doi: 10.1124/dmd.105.007765, PMID: 16381666

[35] Sotaniemi EA Arranto AJ Pelkonen O Pasanen M. Age and cytochrome P450-linked drug metabolism in humans: an analysis of 226 subjects with equal histopathologic conditions*. Clin Pharmacol Ther. (1997) 61:331–9. doi: 10.1016/S0009-9236(97)90166-1, PMID: 9091249

[36] Cotreau MM von Moltke LL Greenblatt DJ. The influence of age and sex on the clearance of cytochrome P450 3A substrates. Clin Pharmacokinet. (2005) 44:33–60. doi: 10.2165/00003088-200544010-00002, PMID: 15634031

[37] Shi Y Au JS-K Thongprasert S Srinivasan S Tsai C-M Khoa MT. A prospective, molecular epidemiology study of EGFR mutations in Asian patients with advanced non–small-cell lung cancer of adenocarcinoma histology (PIONEER). J Thorac Oncol. (2014) 9:154–62. doi: 10.1097/JTO.0000000000000033, PMID: 24419411 PMC4132036

[38] Mendelsohn J Baselga J. Status of epidermal growth factor receptor antagonists in the biology and treatment of cancer. J Clin Oncol. (2003) 21:2787–99. doi: 10.1200/JCO.2003.01.504 12860957

[39] RJCLC P-S. Rash as a surrogate marker for efficacy of epidermal growth factor receptor inhibitors in lung cancer. Clin Lung Cancer. (2006) 8:S7–S14. doi: 10.3816/CLC.2006.s.008, PMID: 17239291

[40] Pastore S Mascia F Mariotti F Dattilo C Mariani V Girolomoni G. ERK1/2 regulates epidermal chemokine expression and skin inflammation. J Immunol. (2005) 174:5047–56. doi: 10.4049/jimmunol.174.8.5047, PMID: 15814736

[41] Tanaka T Matsuoka M Sutani A Gemma A Maemondo M Inoue A. Frequency of and variables associated with the EGFR mutation and its subtypes. Int J Cancer Suppl. (2010) 126:651–5. doi: 10.1002/ijc.24746 19609951

[42] Kari C Chan TO Rocha de Quadros M UJCR R. Targeting the epidermal growth factor receptor in cancer: apoptosis takes center stage. Cancer Res. (2003) 63:1–5. 12517767

[43] Vallböhmer D Zhang W Gordon M Yang DY Yun J Press OA. Molecular determinants of Cetuximab efficacy. J Clin Oncol. (2005) 23:3536–44. doi: 10.1200/JCO.2005.09.100 15908664

[44] Pérez-Soler R Delord JP Halpern A Kelly K Krueger J Sureda BM. HER1/EGFR inhibitor-associated rash: future directions for management and investigation outcomes from the HER1/EGFR inhibitor rash management forum. Oncologist. (2005) 10:345–56. doi: 10.1634/theoncologist.10-5-345, PMID: 15851793

