# Supplementary material for: Dermatologic toxicities in epidermal growth factor receptor: a comprehensive pharmacovigilance study from 2013 to 2023
Source: Front Med (Lausanne). 2024 Jan 24;10:1283807. doi: 10.3389/fmed.2023.1283807 (PMC10848916; doi:10.3389/fmed.2023.1283807)
Supplement: Supplementary file 1 [file Table_1.DOCX]

S1 Table

| Drug name | Entry name |
| --- | --- |
| Afatinib | (2E)-N-(4-(3-Chloro-4-fluoroanilino)-7-(((3S)-oxolan-3-yl)oxy)quinoxazolin-6-yl)-4-(dimethylamino)but-2-enamide; BIBW-2992-MA2; BIBW 2992 MA2; BIBW-2992MA2; BIBW 2992MA2; BIBW2992 MA2; Afatinib Maleate; BIBW 2992; BIBW2992; BIBW-2992;Gilotrif;Afatinib Dimaleate |
| Cetuximab | Erbitux; IMC C225; IMC-C225; MAb C225; C225 |
| Dacomitinib | Vizimpro; N-(4-(3-chloro-4-fluoroanilino)-7-methoxy-6-quinazolinyl)-4-(1-piperidinyl)-2-butenamide; PF 00299804 ;PF00299804; PF-00299804 |
| Erlotinib | Hydrochloride, Erlotinib; Erlotinib HCl; HCl, Erlotinib  ; OSI-774; OSI 774; OSI774; CP 358774 ;358774, CP  ; CP 358,774; 358,774, CP; CP-358,774; CP358,774  ; CP-358774; CP358774; 11C-erlotinib; 11C erlotinib  ; Erlotinib; N-(3-ethynylphenyl)-6,7-bis(2-methoxyethoxy)quinazolin-4-amine; Tarceva |
| Gefitinib | N-(3-Chloro-4-fluorophenyl)-7-methoxy-6-(3-(4-morpholinyl)propoxy)-4-quinazolinamide; Iressa; ZD1839; ZD 1839 |
| Lapatinib | N-(3-chloro-4-(((3-fluorobenzyl)oxy)phenyl)-6-(5-(((2-methylsulfonyl)ethyl)amino)methyl) -2-furyl)-4-quinazolinamine; Tykerb; GW 282974X; GW282974X; GW-282974X; GW572016; GW-572016; GW 572016; Lapatinib Ditosylate |
| Necitumumab | Portrazza; IMC-11F8; 11F8; IMC-11F8 monoclonal antibody |
| Osimertinib | N-(2-((2-(dimethylamino)ethyl)methylamino)-4-methoxy-5-((4-(1-methyl-1H-indol-3-yl)-2-pyrimidinyl)amino)phenyl)-2-propenamide; mereletinib; osimertinib mesylate; osimertinib mesylate ; mereletinib mesylate; N-(2-((2-(dimethylamino)ethyl)methylamino)-4-methoxy-5-((4-(1-methyl-1H-indol-3-yl)-2-pyrimidinyl)amino)phenyl)-2-propenamide methanesulfonate (1:1); AZD9291 mesylate; mereletinib mesylate; AZD-9291 mesylate; AZD9291; AZD-9291; Tagrisso |
| Panitumumab | Panitumumab Antibody, Human; Human Panitumumab Antibody; ABX-EGF Mab; ABX-EGF Monoclonal Antibody; ABX EGF Monoclonal Antibody; Monoclonal Antibody, ABX-EGF; Vectibix |
| Vandetanib | N-(4-bromo-2-fluorophenyl)-6-methoxy-7-((1-methylpiperidin-4-yl)methoxy)quinazolin-4-amine; ZD 6474; ZD6474; ZD-6474; ZD-64; Caprelsa; Zactima |
